# Why Are Sensory Axons More Vulnerable for Ischemia than Motor Axons?

**DOI:** 10.1371/journal.pone.0067113

**Published:** 2013-06-20

**Authors:** Jeannette Hofmeijer, Hessel Franssen, Leonard J. van Schelven, Michel J. A. M. van Putten

**Affiliations:** 1 Clinical Neurophysiology, MIRA Institute for Biomedical Technology and Technical Medicine, University of Twente, Enschede, The Netherlands; 2 Department of Neurology, Rijnstate Hospital, Arnhem, The Netherlands; 3 Department of Neurology and Rudolf Magnus Institute of Neuroscience, University Medical Center Utrecht, Utrecht, The Netherlands; 4 Department of Clinical Neurophysiology, Medisch Spectrum Twente, Enschede, The Netherlands; Albany Medical College, United States of America

## Abstract

**Objective:**

In common peripheral neuropathies, sensory symptoms usually prevail over motor symptoms. This predominance of sensory symptoms may result from higher sensitivity of sensory axons to ischemia.

**Methods:**

We measured median nerve compound sensory action potentials (CSAPs), compound muscle action potentials (CMAPs), and excitability indices in five healthy subjects during forearm ischemia lasting up to disappearance of both CSAPs and CMAPs.

**Results:**

Ischemia induced: (1) earlier disappearance of CSAPs than CMAPs (mean ± standard deviation 30±5 vs. 46±6 minutes), (2) initial changes compatible with axonal depolarization on excitability testing (decrease in threshold, increase in strength duration time constant (SDTC) and refractory period, and decrease in absolute superexcitability) which were all more prominent in sensory than in motor axons, and (3) a subsequent decrease of SDTC reflecting a decrease in persistent Na^+^ conductance during continuing depolarisation.

**Interpretation:**

Our study shows that peripheral sensory axons are more vulnerable for ischemia than motor axons, with faster inexcitability during ischemia. Excitability studies during ischemia showed that this was associated with faster depolarization and faster persistent Na^+^ channel inactivation in sensory than in motor axons. These findings might be attributed to differences in ion channel composition between sensory and motor axons and may contribute to the predominance of sensory over motor symptoms in common peripheral neuropathies.

## Introduction

In common peripheral neuropathies, such as symmetric diabetic neuropathy and chronic idiopathic axonal neuropathy, sensory symptoms usually prevail over motor symptoms [1], [2]. The mechanisms responsible for this discrepancy are unknown. Since nerve ischemia presumably plays a role in the pathophysiology of these neuropathies [3], [4], the predominance of sensory symptoms may result from higher sensitivity of sensory axons to ischemia.

Selective vulnerability to ischemia of sensory relative to motor nerves is supported by several observations. First, nerve conduction studies *in vitro* showed that sensory responses decreased more and recovered less than motor responses during and after episodes of hyperglycemic hypoxia in rats [5]. In human axons, ischemia of 30 minutes duration resulted in disappearance of sensory responses [6], [7] whereas in another study motor responses remained preserved after 25 minutes of ischemia [8]. Second, excitability studies in humans (which assess changes in membrane potential and ion channel activity at one site of a nerve [9]) indicated that ischemia induces more prominent axonal depolarization in sensory than motor axons and that ion channel activity differs between sensory and motor axons [10]–[12].

However, in previous nerve conduction studies, the evolution of sensory and motor responses has not been compared during ischemia. Excitability was investigated during ischemia of 13 [10], 15–20 [13] up to 25 [8] minutes in which period only small decreases in motor [13] [8] or sensory [10] nerve responses of up to 10% were observed. The present study compared the evolution of sensory and motor nerve responses during ischemia lasting up to disappearance of both sensory and motor responses and assessed excitability until just before responses completely disappeared.

## Methods

### Subjects

Experiments were performed on healthy subjects including the authors (Subject (S) 1, female, 33 years; S2, female, 40 years; S3, male, 44 years; S4, male, 49 years; S5, male, 61 years) with a minimal interval of a week between successive experiments on the same subject. The protocol was approved by the institutional review board (Medisch Ethische Toetsingscommissie Twente) and written informed consent was obtained.

### Nerve conduction studies

The median nerve was stimulated by surface electrodes with the cathode at the wrist and the anode four centimetres proximal for both sensory and motor measurements. Compound sensory action potentials (CSAPs) were recorded antidromically by ring electrodes around the distal and proximal interphalangeal joints of the third finger. Compound muscle action potentials (CMAPs) were recorded by surface electrodes from the thenar eminence, with the active electrode at the abductor pollicis brevis muscle and the reference on the proximal phalanx.

During the nerve conduction experiments CSAP and CMAP variables were stored on separate channels, one for sensory and one for motor axons. Measurements included baseline negative peak amplitude, distal sensory latency (DSL), and distal motor latency (DML). Stimuli were single, supra-maximal 1.0 ms duration pulses, delivered once per minute, and nerve conduction variables were obtained every minute. After three minutes, ischemia was induced by a sphygmomanometer cuff around the upper arm, inflated to >200 mmHg. Ischemia was maintained until disappearance of both CSAP and CMAP, or for a maximum duration of one hour.

### Excitability studies

Excitability studies assess passive and active properties of the axon membrane in a limited number of axons at one point of a nerve. At this point, conditioning and test stimuli are delivered. Different types of conditioning are used to change the condition of these axons, e.g., slight subthreshold depolarization, hyperpolarization, or supramaximal stimuli causing Na^+^-channel inactivation. The magnitude of these changes is assessed by test-stimuli which determine threshold during or after a conditioning stimulus. Threshold is the test-stimulus current needed to evoke a nerve response of given magnitude, e.g., a CMAP of 40% of maximal. When a nerve is less excitable, e.g. due to hyperpolarization, threshold is increased and vice versa.

The passive properties that can be assessed by excitability studies, reflect capacitative currents to and from the axolemma which change membrane potential. For instance, outward capacitative currents accumulate positive ions on the inside of the axolemma and depolarize membrane potential. Membrane potential changes, induced by capacitative currents, are fast for the tiny nodal surface but slow for the large internodal surface of the axolemma. Active properties reflect ionic currents passing through open voltage-gated ion-channels in the axolemma. The nodal axolemma contains transient Na^+^-channels, persistent Na^+^-channels, and slow K^+^-channels. The internodal axolemma contains fast and slow K^+^-channels, Na^+^/K^+^-pumps, and hyperpolarization-activated-nucleotide (HCN)-channels. Under physiological conditions, Na^+^-channels pass an inward ionic current that changes membrane potential in a depolarizing direction, whereas K^+^-channels pass an outward ionic current that changes membrane potential in a hyperpolarizing direction. Transient Na^+^-channels open briefly on depolarization and inactivate thereafter; they underlie action potential generation. Persistent Na^+^-channels remain open over a wide range of membrane potential values and are more expressed in sensory nodes. Fast K^+^-channels open rapidly on depolarization and oppose spreading of the action potential to the internode. Slow K^+^-channels open on long standing depolarization. HCN-channels open on long standing and prominent hyperpolarization, passing an inward current that changes membrane potential in depolarizing direction.

In the present study, excitability tests were performed to reveal changes in median nerve motor and sensory axons at the wrist during ischemia and in the post-ischemic period. In previous studies, fast excitability protocols have been developed that assess a limited number of excitability-indices at short time intervals, ranging from 5 - 60 seconds [10]. For two reasons the time interval between successive excitability-tests was approximately 5 minutes in our experiments. First, since we found that CSAP and CMAP amplitudes changed during longer lasting ischemia, each excitability-test had to include a stimulus-response curve in order to estimate baseline-threshold as accurately as possible. Second, we were interested in changes in membrane potential, transient Na^+^-current, persistent Na^+^-current, and slow K^+^-current. Therefore, each excitability-test had to include stimulus-response curve, strength-duration properties, and recovery cycle. Since this protocol took about 4–5 minutes to perform, the interval between the start of successive tests was 5 minutes. Threshold electrotonus was omitted because it would have taken too much time.

Separate excitability-tests were performed for motor and sensory axons. Threshold was defined as a target-response (CSAP or CMAP) of 40% of its maximal amplitude, so that myelinated axons with intermediate threshold and intermediate diameter were investigated. Threshold was determined by 1.0 ms duration test-stimuli using computerized proportional tracking. Threshold changes were induced by conditioning current stimuli. The QTRAC and TRONDXM programs controlled conditioning and test stimuli (developed by prof. H. Bostock, Institute of Neurology, Queen Square, London) [14].

Stimulus-response curve was recorded to 1.0 ms duration stimuli. It was used to determine baseline threshold for the target-response and to facilitate proportional threshold tracking because it yields the change in target-response amplitude per current step.

Strength-duration properties were determined by tracking the current for the target-response for stimuli of different duration (0.2, 0.4, 0.6, 0.8, and 1.0 ms). Per stimulus, stimulus-current was converted to stimulus-charge by multiplying stimulus-current with stimulus-duration, thereby yielding a charge-duration plot. The charge-duration relation is linear, with the slope representing rheobase and the x-intercept representing strength-duration time constant (SDTC). Rheobase is the smallest current necessary for a target response if stimulus duration is infinitely long. SDTC reflects persistent nodal Na^+^-current and the time constant for charging the nodal membrane. As the passive membrane properties are not likely to change in our experiment, any increase in SDTC will reflect an increase in persistent nodal Na^+^-current [15].

Recovery cycle was recorded by giving a supramaximal 1.0 ms duration conditioning stimulus followed by a test stimulus tracking the threshold for the target CSAP or CMAP. Conditioning-test intervals ranged from 200 ms to 2 ms. CSAPs and CMAPs for threshold estimation were derived on line by subtracting the CSAP or CMAP to a single supramaximal stimulus from the overlapping CSAPs or CMAPs to the conditioning and test stimulus pair. We determined (i) refractory period: the time between test stimulus and the moment that threshold has returned to normal. This reflects transient Na^+^-current inactivation after the action potential [16]. (ii) 50% refractory period: the time between the test stimulus and the moment that threshold has returned to 50% above its normal value, (iii) superexcitability: the lowest threshold after refractory period, reflecting the depolarizing afterpotential [17]. and (iv) subexcitability: the highest threshold after superexcitability, reflecting activation of slow K^+^-current by the depolarizing afterpotential. The depolarizing afterpotential in myelinated axons arises because the inward current of the action potential causes a capacitative outward current across the internodal membrane that charges its inside; subsequently this charge dissipates to the node, causing the depolarizing afterpotential [17].

### Experimental protocol per subject

Nerve conduction studies to obtain CMAP and CSAP values, excitability studies of sensory axons, and excitability studies of motor axons were performed in each subject during three separate experiments with a minimal interval of a week between successive experiments on the same subject. The experimental protocol for a subject consisted of: nerve conduction or excitability-tests in the pre-ischemic period, start of ischemia by inflation of a cuff around the upper arm to a pressure of 200 mm Hg, successive excitability tests until the CSAP or CMAP decreased to such an extent that responses could no longer be obtained, release of ischemia, and nerve conduction or excitability tests five minutes after the release of ischemia. Nerve conduction variables were obtained every minute. Each excitability test lasted about five to ten minutes. The interval between excitability tests was five minutes. Temperature was measured at the wrist, adjacent to the stimulating cathode, by a laser radiator (Comark Limited, KM814).

During the entire protocol, the hand and fingers were fixed with adhesive tape in order to avoid artefacts due to gradual changes in hand or limb position. Both stimulation and recording electrodes were carefully fixed and controlled by the experimenter (HF or JH) who was continuously present and who also kept the level of cuff inflation at the same value.

### Analysis

Nerve conduction and excitability variables before the induction of ischemia are presented as mean ± standard deviation (SD) for sensory and motor nerves. Variables during and after ischemia are normalized to pre-ischemic levels for each subject and mean normalized values are presented. Differences between sensory and motor responses are analyzed with Student's t-test and presented as mean differences (MD)±95% confidence intervals (CI).

## Results

### Clinical symptoms

All subjects in all experiments experienced paresthesia during the first ten to fifteen minutes of ischemia, followed by progressive numbness up to complete anesthesia with disturbed proprioception. Reperfusion was followed by severe parestesia and involuntary movements of the hand and fingers during several minutes, followed by mild parestesia during several hours. Fasciculations were not observed.

### Nerve conduction studies

**Figure 1:**
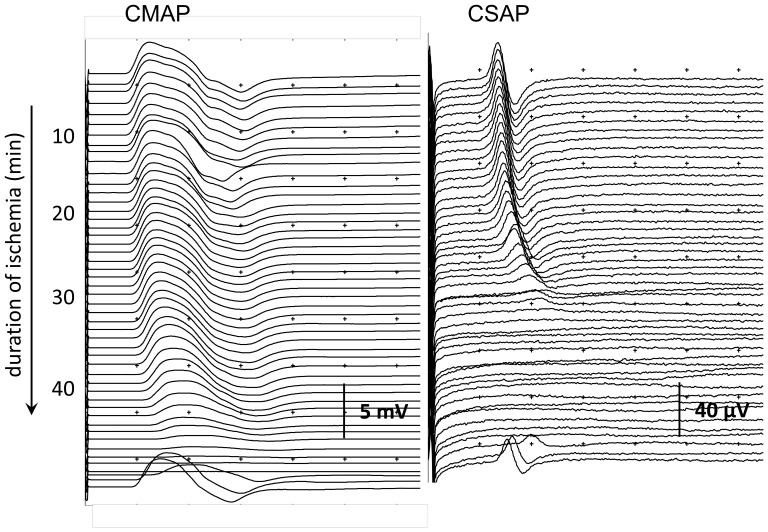
Evolution of compound motor action potential (CMAP) and compound sensory action potential (CSAP) during and after ischemia in one subject. The CMAP disappeared after 44 minutes. The CSAP disappeared after 28 minutes. Reperfusion was started immediately after disappearance of the CMAP, with prompt reversal of CMAP and CSAP.

**Figure 2:**
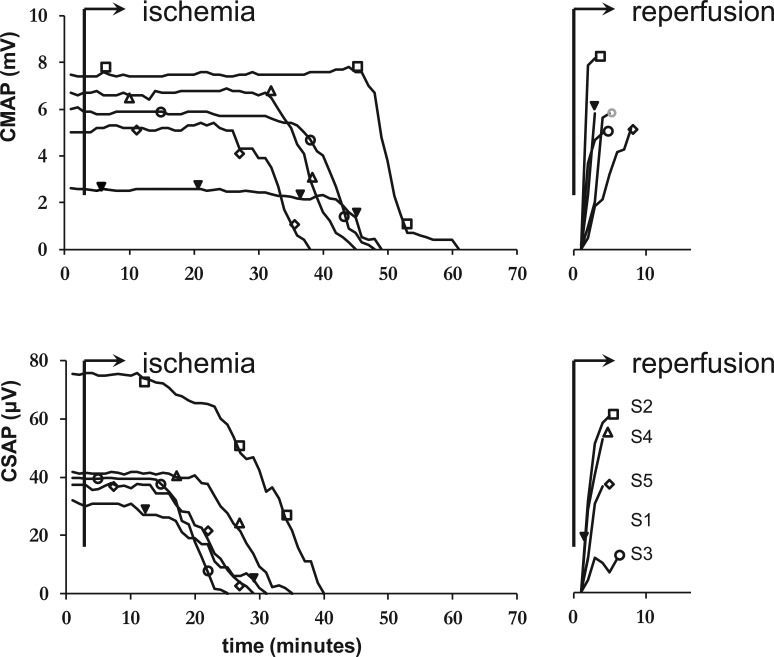
CMAP and CSAP amplitudes of median nerve axons after stimulation at the wrist in five healthy subjects. Ischemia was induced after three minutes and lasted until disappearance of the CMAP in all subjects. Inexcitability occurred earlier in sensory than in motor nerves, reflected as disappearance of the CMAP after 46±6 minutes and the CSAP after 30±5 (mean difference 15 minutes, 95% CI 7–24). CMAP and CSAP were measured every minute and different symbols indicate different subjects: Subject (S) 1  =  female, 33 years; S2  =  female, 40 years; S3 = male, 43 years; S4  =  male, 49 years; S5 =  male 61 years.

Nerve conduction studies were performed in five subjects. The results are presented in Table 1 and Figures 1 and 2. Ischemia lasted up to disappearance of the CMAP in all subjects. After the onset of ischemia, CSAP amplitude started to decrease earlier than CMAP amplitude: CSAP amplitude was reduced by 20% after 18±4 minutes and CMAP amplitude was reduced by 20% after 37±4 minutes (MD 19 minutes, 95% CI 12–25). Complete disappearance of responses occurred after 30±5 minutes for CSAPs and after 46±6 minutes for CMAPs (MD 15 minutes, 95% CI 7–24). However, once responses started to decrease, CMAPs decreased faster than CSAPs; the time interval between 20% and 80% reduction in amplitude was 9±3 minutes for CSAPs and 5±2 minutes for CMAPs (MD 4 minutes, 95% CI 0–7). DSL increased from 3.0±0.1 ms at the onset of ischemia to 5.1±0.4 ms one minute before disappearance of the response. DML increased from 3.6±0.2 ms to 8.0±1.5 ms. The increase of DML was larger than the increase of DSL (125±35% vs. 70±14%, MD 55%, 95% CI 16–93).

**Table 1:** Nerve conduction and excitability variables before, during and after ischemia.

		Motor measurements							Sensory measurements					Mean Difference (95% CI) between motor and sensory nerves of early (Isch1) changes during ischemia	
	Before	Isch1	Isch2	Isch3	Isch4	Isch5	After	Before	Isch1	Isch2	Isch3	Isch4	Isch5	After	
***Conduction variables***															
Amplitude (mV/µV)	5.3±2.0	−4%	−4%	−3%	−7%	−26%	−4%	41.2±15.3	0%	−7%	−40%	−67%	-	−44%	
Latency (ms)	3.5±0.2	14%	12%	23%	41%	59%	35%	3.0±0.1	6%	12%	22%	37%	-	28%	
***Stimulus response curve***															
threshold	6.9±3.2	−8%	−12%	−18%	−16%	5%	26%	5.8±2.5	−15%	−10%	−10%	-	-	25%	7 (0 to 15)
***Strength-duration properties***															
Rheobase (mA)	4.7±2.4	−14%	−9%	−17%	−2%	39%	43%	2.8±1.2	−32%	−13%			-	5%	19 (8 to 29)
SDTC (ms)	0.50±0.08	7%	−5%	−2%	−9%	−18%	−33%	0.55±0.10	25%	12%		-	-	−0%	19 (−1 to 38)
***Recovery cycle***															
50% refractory period (ms)	2.7±0.3	30%	70%	179%	240%	315%	−10%	2.0±0.6	130%	260%	-	-	-	30%	101 (81 to 120)
Super-excitability (%)	−26±3	−79%	−89%	-	-	-	77%	−17±6	-	-	-	-	-	61%	20 (−3 to 38)
Sub-excitability (%)	21±10	4%	−40%	-	-	-	−29%	13±6	-	-	-	-	-	−23%	29 (−122 to 180)

Variables before induction of ischemia are presented as mean values ± standard deviation. Values during and after ischemia are expressed as a percentage of values before ischemia with negative values indicating a decrease and positive values an increase. Nerve conduction variables were obtained every minute. One excitability-test lasted about five to ten minutes. The interval between excitability-tests was five minutes. At Isch 5 (from 45 tot 50 minutes after the induction of ischemia) in none of the subjects any sensory response could be measured any more. CI  =  confidence interval; Before  =  before induction of ischemia; Isch1  =  from 5 to 10 minutes after the induction of ischemia; Isch2 = 15–20 minutes; Isch3 = 25–30 minutes; Isch4 = 35–40 minutes; Isch5 = 45–50 minutes; after  = 5–10 minutes after release of ischemia; -  =  variable not measurable; SDTC  =  strength duration time constant.

### Excitability studies

Excitability studies were performed in four subjects (S2, S3, S4, and S5). For all analyses, 50% refractory period instead of refractory period was used, since the latter could frequently not be measured as a result of high threshold values.

Before ischemia was induced, sensory axons had lower rheobase and higher SDTC than motor axons, a combination that is consistent with the well-known more prominent persistent Na^+^-current in sensory axons (Table 1) [15]. In sensory axons, 50% refractory period was shorter and superexcitability smaller than in motor axons; this combination suggests that the initial event leading to the recovery cycle (i.e., the action potential) is smaller in sensory axons, which is consistent with previous findings [17].

**Figure 3:**
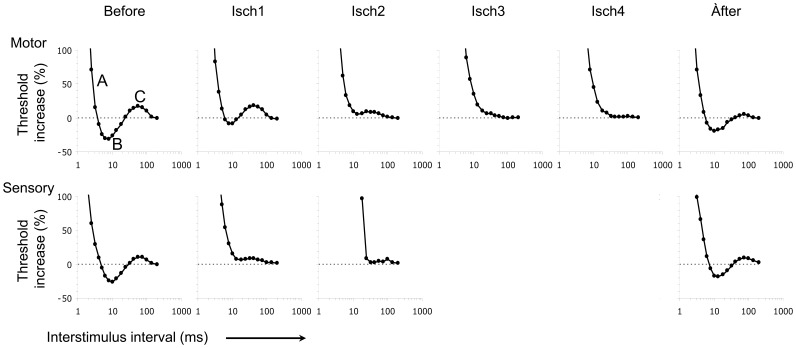
Recovery cycle of motor and sensory median nerve axons stimulated at the wrist before, during, and after ischemia in one subject. Threshold increase is plotted upwards. Before  =  before induction of ischemia; Isch1  =  from 5 to 10 minutes after the induction of ischemia; Isch2 = 15–20 minutes; Isch3 = 25–30 minutes; Isch4 = 35–40 minutes; Isch5 = 45–50 minutes; after = 5–10 minutes after release of ischemia; A = 0% refractory period; Time at first x-axis intercept  =  refractory period; B  =  superexcitability; C  =  subexcitability.

**Figure 4:**
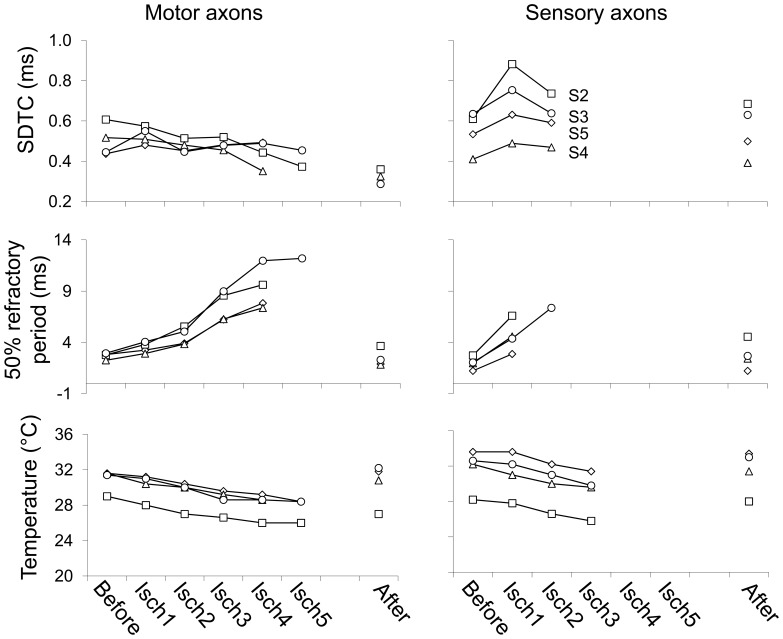
Strength duration time constant (SDTC), 50% refractory period, and temperature for motor and sensory axons before, during and after ischemia in four healthy subjects. Before  =  before induction of ischemia; Isch1  =  from 5 to 10 minutes after the induction of ischemia; Isch2 = 15–20 minutes; Isch3 = 25–30 minutes; Isch4 = 35–40 minutes; Isch5 = 45–50 minutes; after = 5–10 minutes after release of ischemia. Subject (S) 2 = female, 40 years; S3 = male, 43 years; S4 = male, 49 years; S5 = male 61 years.

During ischemia, the following changes were found in both sensory and motor excitability indices (Table 1, Figures 3 and 4). Rheobase decreased and SDTC increased, indicating that axons were more excitable due to an increase in persistent Na^+^-current. The decrease in threshold is consistent with this. Furthermore, 50% refractory period increased, indicating an increase of transient Na^+^-channel inactivation. Finally, absolute super- and subexcitibility values decreased, probably as a result of the decrease in Na^+^-influx due to transient Na^+^-channel inactivation. These findings suggest that ischemia induced depolarization that resulted in an increased persistent Na^+^-current and inactivated transient Na^+^-current.

In ischemia lasting more than approximately twenty to thirty minutes, the initial decrease in rheobase and threshold was followed by a subsequent gradual increase, and the increase in SDTC was followed by a decrease after approximately ten minutes (Table 1). The subsequent gradual decrease in SDTC reflects a decrease in Na^+^ conductance during continuing depolarisation. The continuous decrease of 50% refractory period is consistent with this. Absolute super- and subexcitibility values decreased to such an amount, that it was no longer possible to quantify these after 5–10 minutes for sensory and 25–30 minutes for motor measurements.

After ischemia, we found changes in the opposite direction for both sensory and motor responses. Threshold and rheobase increased and SDTC decreased, indicating that axons were less excitable. The increase of absolute superexcitability values is consistent with this. These findings suggest that reperfusion induced hyperpolarization that resulted in a decrease in persistent Na^+^-current (Table 1).

In sensory axons, ischemia induced a larger initial decrease in threshold and rheobase, and a larger initial increase in SDTC, indicating a larger increase of excitability due to a larger increase in persistent Na^+^-current. Furthermore, there was a larger increase of 50% refractory period in sensory axons, indicating more prominent transient Na^+^-channel inactivation. Finally, there was a faster decrease of absolute super- and subexcitibility values, presumambly reflecting the faster decrease in threshold of sensory axons.

### Temperature

Temperature during the experiments is presented in Figure 4. Temperature decreased during ischemia from 31.3±2.4°C to 28.0±2.2°C during sensory and from 31.1±1.9°C to 27.4±1.4°C during motor measurements. This indicates a 10.4±3.1% decrease during sensory and an 11.6±2.1% decrease during motor measurements (MD 1.2%, 95% CI −1.8 to 4.2). This difference between sensory and motor measurements was not statistically significant.

## Discussion

For the first time we compared nerve conduction and excitability variables of peripheral sensory and motor axons during ischemia lasting up to attenuation and disappearance of both motor and sensory responses. CSAPs disappeared after 30±5 minutes, followed by CMAPs after 46±6 minutes. Excitability studies showed that ischemia induced an initial decrease in threshold, an initial increase in SDTC, and an increase in 50% refractory period. The largest changes of threshold and SDTC occurred within the first ten to fifteen minutes of ischemia and were more prominent in sensory axons. Remarkably, the initial increase in SDTC was followed by a decrease and the initial decrease in threshold by an increase.

Previously, sensory axons have been exposed to ischemia for up to 30 minutes showing that, after this period, CSAPs were unobtainable when recorded by surface electrodes, but preserved, although attenuated, when recorded by near-nerve needle electrodes [6], [7]. In another study, motor axons were exposed to ischemia for up to 25 minutes, which did not result in attenuation of CMAPs [8]. However, a head to head comparison between the evolution of CSAPs and CMAPs in the same subject during ischemia, showing that CSAPs are unobtainable sooner than CMAPs, has not been performed. The effects of ischemia on excitability of sensory and motor axons has been studied before [12], [18], but only one study focussed on comparing excitability indices between human sensory and motor nerves during ischemia lasting up to thirteen minutes. This showed that both ischemic depolarization and post-ischemic hyperpolarization were greater for sensory than motor axons [10]. Our results are in agreement with this report. In addition, we showed that the changes of threshold and SDTC indeed occur within the first ten to fifteen minutes after the onset of ischemia[19] and thereafter slightly reverse. Otherwise, 50% refractory period continuously increased up to complete disappearance of the responses.

Our findings before the induction of ischemia are consistent with previous reports on biophysical differences between sensory and motor axons at rest [15], [17]. These include lower rheobase, larger SDTC, shorter refractoriness, and smaller absolute superexcitability for sensory axons which were attributed to larger persistent Na^+^-current [20] and smaller action potentials in sensory axons. Recent observations suggest that the larger persistent Na^+^-current in sensory axons does not result from greater expression of persistent Na^+^-channels, but to a more depolarized membrane potential, resulting in a larger difference with the sodium Nernst potential, causing a larger persistent sodium current [21]. The larger persistent Na^+^ current in sensory axons results in a stronger dependency on the Na^+^-K^+^ pump to maintain physiological ion gradients, which can explain the larger sensitivity to ischemia. During ischemia, failure of this energy-dependent pump may then result in faster decrease of transmembrane ion gradients and depolarization in sensory axons. This is in agreement with the initial changes we observed during ischemia: decreased threshold, increased SDTC, and increased refractory period, which have been shown to be associated with membrane depolarization [8], [22] and were more prominent in sensory axons.

When ischemia continued, the initial increase in SDTC was followed by a decrease, as noted previously in sensory axons after eighteen to twenty minutes of ischemia [23]. The initial increase in SDTC likely reflects an increase in persistent Na^+^ current due to ischemic depolarization [15], [21]. The subsequent gradual decrease in SDTC reflects a decrease in Na^+^ conductance during continuing depolarisation.

The continuous increase in 50% refractory period during ischemia is consistent with gradually developing transient Na^+^ current inactivation due to continuing depolarization [10], [24]. For 50% refractory period these changes were more prominent in sensory axons, again indicating faster inactivation of transient Na^+^ channels in sensory than motor axons due to more prominent depolarization.

CMAPs remained unchanged relatively long. However, once responses started to decrease, CMAPs decreased faster than CSAPs. A possible explanation might be that impulse transmission from the main motor axon to terminal axon branches remains stable over a wide range of reduced impulses in the main axon, but becomes compromised if the impulse in the main axon decreases below a certain value.

In previous reports, rapid changes of excitability parameters, with fluctuations beyond one hour after ischemia of five to ten minutes have been shown.[13], [25] We found changes shortly after the release of ischemia, which were qualitatively similar for both sensory and motor axons and included a decrease in threshold and increase in SDTC. However, our measurements after release of ischemia were of too short duration, and each excitability measurement lasted too long, for a valid discussion of responses after reperfusion, including differences between sensory and motor axons.

Selective ischemic vulnerability has also been attributed to morphological properties. Both in healthy animals [26], [27] and in patients with vasculitic neuropathy [28] myelinated large fibers were found to be more vulnerable than unmyelinated small fibers [26], [26]–[28]. Also, the density and spatial distribution of capillaries has been associated with sensitivity to ischemia, with a higher ischemic vulnerability if endoneurial capillary density was lower, probably indicating that ischemic vulnerability is lower if the vascular reserve capacity is larger [29], [30]. However, no differences with regard to these parameters were found between sensory and motor axons.

Excitability studies in patients with diabetic neuropathy showed a decreased SDTC, ascribed to a reduction of nodal Na^+^-current, and a slower recovery of activity-induced threshold increase, ascribed to Na^+^-K^+^ pump dysfunction [31]. Furthermore, alterations in the recovery cycle were observed (decreased refractory period, superexcitability, and subexcitability), which were ascribed to smaller action potentials due to reduced nodal Na^+^ current [4]. On the other hand, although nerve ischemia probably plays a role in the pathophysiology of diabetic neuropathy, the presence of diabetes mellitus may protect against the consequences of nerve ischemia, as sensory and motor responses were less attenuated during ischemia in diabetic patients than in healthy subjects [32]–[34]. This has been ascribed to a reduction of nodal Na^+^ channels with smaller Na^+^ currents in diabetic neuropathy [4], [35].

Our study has some limitations. First, we measured effects of acute transient ischemia, whereas, if nerve ischemia plays a role in the pathophysiology of diabetic neuropathy or chronic idiopatic axonal neuropathy, this would most probably be chronic mild ischemia. However, Huynh and colleagues have reported on similar findings on excitability studies in a patient with chronic limb ischemia [36]. Second, temperature decreased during the experiments and in a previous study we found that cooling induces changes compatible with axonal depolarization as well [37], [38]. However, the excitability indices in the present study changed far more prominently than can be expected from the observed decrease in temperature alone. In the present study, temperature decreased approximately 10%, with an increase of SDTC of 25% and 50% refractory period of 315%. In our previous study, even a 10-fold decrease in temperature without ischemia did not induce excitability changes to such extent [37], [38]. Furthermore, we found no statistically significant differences in temperature decrease between sensory and motor nerves. Third, changes in CMAP amplitude could reflect ischemic changes in muscle. However, since isolated muscle ischemia induces a reduction in CMAP amplitude, the earlier disappearance of CSAPs than CMAPs in our study cannot be attributed to effects of muscle ischemia [39]. Fourth, due to our choice for a relative long-duration excitability protocol, the time-resolution for changes preceding the development of inexcitability was less favorable than when multitrack protocols would have been used. Multitrack protocols assess a more limited number of excitability-indices with a high frequency, for example every 5 seconds [10]. Fifth, we cannot completely exclude the effects of temporal dispersion. It has been found that temporal dispersion is an important factor underlying CSAP amplitude reduction during the first 20 minutes of ischemia [40] and its effects are probably larger on CSAP than on CMAP amplitudes [41]. However, according to figure 1, it seems unlikely that temporal dispersion was a major factor causing the quicker reduction in CSAP amplitude during ischemia in our study, although we cannot exclude the effects of phase cancellation. Finally, we did not measure serum or tissue pH or any other indicator of metabolic derangement. Threshold fluctuations have been associated with tissue acidosis in patients with diabetes mellitus [42] and in patients with critical illness polyneuropathy multiple excitability indices of membrane potential were related to respiratory acidosis [43]. However, it is unlikely that our observed changes resulted from changes of pH, since acidosis has been associated with threshold increase and SDTC decrease [44].

## Conclusion

We demonstrated that during ischemia CSAPs were unobtainable sooner than CMAPs. This was associated with faster depolarization and faster Na^+^ channel inactivation in sensory axons. Our findings may contribute to the understanding of the predominance of sensory symptoms in common peripheral neuropathies.
